# A Comparison and Integration of MiSeq and MinION Platforms for Sequencing Single Source and Mixed Mitochondrial Genomes

**DOI:** 10.1371/journal.pone.0167600

**Published:** 2016-12-09

**Authors:** Michael R. Lindberg, Sarah E. Schmedes, F. Curtis Hewitt, Jamie L. Haas, Krista L. Ternus, Dana R. Kadavy, Bruce Budowle

**Affiliations:** 1 Signature Science, LLC, Austin, Texas, United States of America; 2 Institute of Applied Genetics, Department of Molecular and Medical Genetics, University of North Texas Health Science Center, Fort Worth, Texas, United States of America; 3 Center of Excellence in Genomic Medicine Research (CEGMR), King Abdulaziz University, Jeddah, Saudi Arabia; Xiamen University, CHINA

## Abstract

Single source and multiple donor (mixed) samples of human mitochondrial DNA were analyzed and compared using the MinION and the MiSeq platforms. A generalized variant detection strategy was employed to provide a cursory framework for evaluating the reliability and accuracy of mitochondrial sequences produced by the MinION. The feasibility of long-read phasing was investigated to establish its efficacy in quantitatively distinguishing and deconvolving individuals in a mixture. Finally, a proof-of-concept was demonstrated by integrating both platforms in a hybrid assembly that leverages solely mixture data to accurately reconstruct full mitochondrial genomes.

## Introduction

High-throughput, or massively parallel, sequencing has been a boon to many fields interested in omics, ranging from basic research to precision medicine to even the forensic sciences. Some technologies now offer the capability of single-molecule sequencing, generating reads averaging several thousand bases in length [1, 2]. The appeal of long, single-molecule sequencing is the potential to determine variant phase along a chromosome, identify copy number variants, determine gene organization, and improve *de novo* sequencing results in an expeditious and cost effective manner. The most recent instrument and chemistry to perform single-molecule sequencing is the MinION™ (Oxford Nanopore Technologies, Oxford, UK) which combines a customized protein nanopore, a sequencing flow cell, and accompanying electronics into a palm-sized device [2]. Two studies have reported the per-base accuracy of sequencing randomly sheared shotgun libraries with MinION R7 and R7.3 chemistries [3, 4]. Ashton et al. [5] showed that the long reads generated by nanopore sequencing could infer gene organization; however, Illumina sequence data were relied upon to construct a scaffold for read mapping. The MinION has been largely used to sequence amplicons [6], whole genomes [3] of bacteria and viruses, and more recently murine and yeast mitochondrial genomes [7–9]. This system has substantial appeal due to generation of long reads, relatively simple sample preparation, flexible run times, small footprint, and portability. However, with all these features there have been few published studies describing its utility outside sequencing microbes. Presumably, the relatively higher error rates and need for data generated to be used in conjunction with lower error rate short-read data limit the application of the MinION to date. There are applications, however, where this chemistry may be useful and may be able to provide analyses on its own, such as analysis of mixtures of the mitochondrial genome where the contributions are phylogenetically the same or similar [10–13]. Interpretation of mixture evidence is critical but challenging in forensic genetics [14–17], but advancements also apply to transplantation monitoring and *de novo* mutation detection in heterogeneous or mosaic mitochondrial populations.

The mitochondrial genome is an ideal molecule to study because its population genetic variance is well-defined; it lacks recombination, and is inherited maternally. Its haploid state, compact size (~16,569 base pairs), and concentration of variation in the control region have made the mitochondrial genome an informative target for numerous applications [11, 18–22]. In particular, the mitochondrial genome is sequenced to identify human remains [23], characterize challenged samples from mass disasters or mass graves [24, 25], establish kinship [26], characterize tainted food products [27–29], assist in wildlife poaching investigations [30, 31], characterize ancient samples [32, 33], and serve as a clinical diagnostic [34]. The high copy number of the mitochondrial genome per cell enhances the chance of typing results in highly degraded samples and as an example was successfully typed from Neanderthal remains [35]. Mitochondrial DNA sequencing is traditionally performed using Sanger sequencing, targeting the two hypervariable regions (HVR1 and HVR2) residing in the non-coding portion of the genome [36, 37]. Although a mainstay methodology, it is laborious and time-consuming, and requires costly sequencing equipment. Another limitation of Sanger sequencing is that samples composed of mixtures cannot be readily deconvolved because the output is not quantitative [38]. Massively parallel sequencing (MPS) has made it possible to expand sequencing to cover the entire mitochondrial genome in a more effective, more quantitative, less laborious, and far less costly manner [12, 39–41]. Moreover, whole-mitochondrial genome sequencing reduces error in haplogroup assignment [40], which in turn improves understanding of the evolutionary history of humans. With samples composed of two or more individuals, quantitative differences, as well as phylogenetically informative sites, can be used to phase certain variants to each contributing genome, as the read lengths, ~300 bps for the MiSeq™ (Illumina, San Diego, CA), are too short to cover multiple informative variants. However, when the amount of DNA from multiple contributors in a sample is comparable and the individuals are phylogenetically similar, deconvolving the haplotypes (i.e., assigning private mutations) is not possible with short reads alone.

Even with its relatively high error rate, it is possible that the MinION system could assign the variant states correctly to contributors (i.e., phasing) of a mixed sample without relying on lower error rate short read MPS generated data from non-degraded samples. In the study herein, well-defined single source mitochondrial genome samples of the U2e1a1 haplogroup were mixed and sequenced blindly to determine the efficacy of the MinION system to accurately characterize the individual contributors of the artificially mixed sample. An unbiased approach was taken to evaluate single nucleotide polymorphisms (SNPs) identified by the MiSeq and MinION sequencers. Using a naïve approach, the variant allele frequency (VAF) (defined as the fraction of reads representing a variant in a heterogeneous (or heteroplasmic) sample) of the MiSeq platform was used to establish a conservative truth set with the intent to limit the number of false positives. Since alignment strategies and chemistries differ between the MiSeq and MinION technologies, it was deemed better to apply a global VAF threshold at the outset of SNP discovery and not to apply strict quality filters to the MiSeq generated data when comparing results. This approach ensured a platform independent, agnostic evaluation where local realignment and filtering of alignment artifacts present in loci of known variation length heteroplasmy compounded by homopolymeric repeats [42, 43]. Putative SNPs located in these regions can introduce both false positives and false negatives into the ground truth. In this study, concordance was determined empirically, resulting in at most one false negative SNP in the ground truth with respect to previous work [12, 40]. These SNPs, while few in number, were all present in loci of length heteroplasmy and do not fundamentally change the findings presented here. The overall results indicate that the MinION system is capable of detecting SNPs on the mitochondrial genome with relatively high accuracy and can correctly phase SNPs in fragments greater than 8000 bases in length (which is the length of the long-PCR amplicons generated) without reliance on MPS data. When combined, both platforms can be used to reconstruct complete mitochondrial assemblies containing all sites of variation for individuals contributing to a mixture.

## Results and Discussion

### Sample Selections and Experimental Design

**Fig 1:**
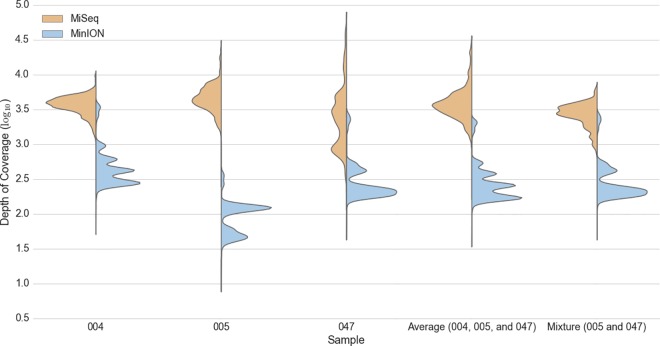
Mitochondrial Depth of Coverage. Coverage distributions of MiSeq (orange) and MinION (blue) sequencing data are grouped together by sample across the x-axis. Depth observations are plotted against the y-axis with a log10 scale. The average distributions stem from the single source samples (004, 005, and 047) only.

The three single source samples (004, 005, and 047) and one mixture (1:1 concentration of 005 and 047) were sequenced on the Illumina MiSeq and Oxford Nanopore Technologies MinION. The samples 005 and 047 were chosen for the mixture because they share the same haplogroup and can only be distinguished by private SNPs with genomic distances greater than typical short-read sequencing workflows. Alignment coverage for the mitochondrial genome in each of the four experiments and the average across the three single source samples are shown in Fig 1. As expected, the MiSeq produced an order of magnitude greater depth of coverage on average than the MinION.

### Single Source Evaluation

**Fig 2:**
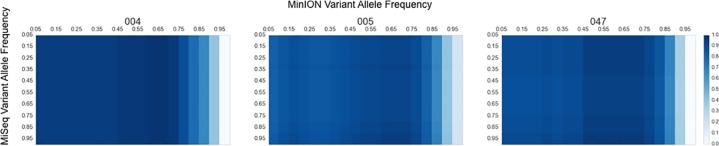
Single Source Sample Concordance by VAF. Each single source sample (004, 005, and 047) is characterized by a heatmap, which compares SNP call sets between the two platforms. SNP call sets are plotted by VAF for both the MinION and MiSeq data from 0.05 to 0.95 using increments of 0.05. The MinION VAF is on the x-axis and the MiSeq VAF is across the y-axis. Concordance is determined by calculating the F1-Score using the MiSeq calls as the ground truth. The value of each F1-Score comparison is shown as increasingly darker shades of blue for higher values. The highest F1-Scores are shown for each of the source samples 004, 005, and 047 in Fig 3, S2 and S3 Figs, respectively.

SNP concordance between the platforms was measured by performing a broad assessment of VAF in each of the three single source samples. The SNPs discovered in the variant calling using the MiSeq data were used as the ground truth. F1-Scores, the harmonic mean of precision and recall, were plotted across the range of VAFs for each individual (Fig 2). The highest observed F1-Score (or highest concordance obtained) between the two platforms across all single source experiments occurred when the VAF for the MiSeq was between 0.90 and 0.95 and the MinION was between 0.60 and 0.65. Even though the VAF analytic thresholds were determined empirically for these platforms, they are reasonable for this approach, given the accepted accuracy rates of the two platforms [44, 45] and the difficulty of detecting low-level heteroplasmy [21]. Putative length heteroplasmy causes an exclusion of a bonafide SNP at np 16,183 in the truth sets of both 005 and 047; however, capturing this SNP at a lower VAF will include this SNP and will include a false positive at np 310 (S1 Fig). It should be noted that differences in substitution and gap penalties of aligners can result in alternate alignments depending on criteria such as alignment start position, read length, and the distribution of mismatches present in the query, which are compounded in repetitive regions when analyzing short reads.

**Fig 3:**
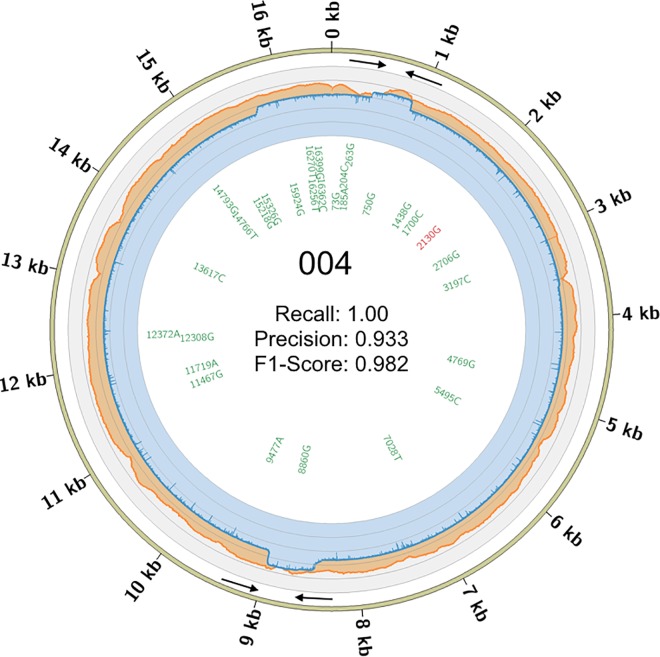
Coverage and Concordance Circos for 004. The coverage depth per base is shown for the MiSeq (orange) and MinION (blue) shown on the outer ring using a log10 scale. The inner ring shows concordance at each SNP using a MiSeq VAF of 0.90 and a MinION VAF of 0.65. Text color denotes the categorization of each SNP under the VAF combination providing the highest concordance (F1-Score). Green text indicates true positives and black text is for false negatives present in the MinION call sets. See S2 and S3 Figs for similar plots of 005 and 047, respectively. Black arrows indicate the locations and orientations of the primers used for amplification.

**Fig 4:**
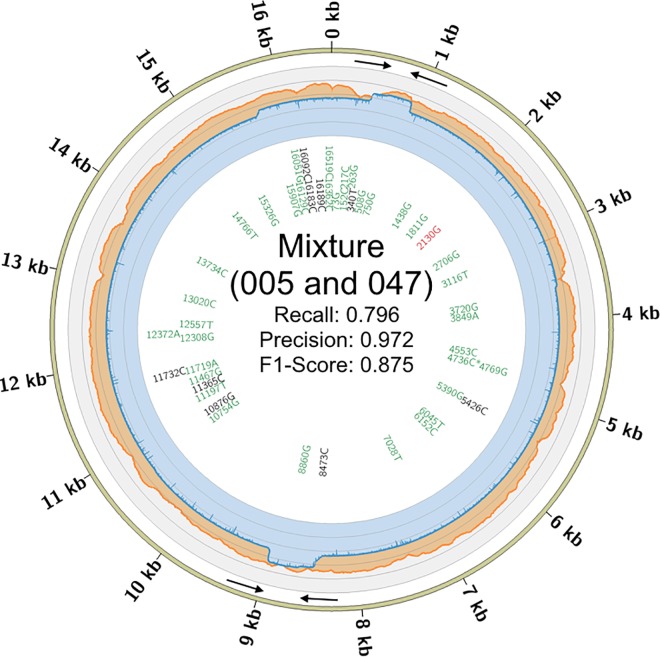
Coverage and Concordance Circos for Mixture (005 and 047). The coverage depth per base is shown for the MiSeq (orange) and MinION (blue) shown on the outer ring using a log10 scale. The inner ring contains the per SNP concordance using a VAF of 0.25 for the MiSeq and 0.45 VAF for the MinION. The text color denotes the categorization of each SNP using a VAF combination providing the highest concordance: green text indicates that the SNP was a true positive, black text is for false negatives, and red is for false positives in the MinION call sets. An asterisk means the SNP was not observed at the MiSeq VAF and not used for calculating recall, precision, and the F1-Score. Black arrows indicate the locations of the primers used for amplification.

A strong overall concordance was observed between the two platforms with F1-scores of 0.982 (TP: 28, FP: 1, FN: 0) 0.946 (TP: 35, FP: 1, FN: 3), and 0.957 (TP: 34, FP: 0, FN: 3) for the three single source samples 004, 005, and 047, respectively. The site-specific agreement per SNP and coverage can be seen in Fig 3, S2 and S3 Figs. False negatives, on both platforms, occurred consistently in the HVRI site (np 16,183–16,189), and particularly with the MinION, which is more refractory to sites containing homopolymeric runs of 5 Cs or longer. A single false positive was observed on the MinION in one dataset (005) in a locus (np 2,130–2,135) that contains 6As in a row. It is not surprising that sequencing through homopolymers of this length is difficult for the MinION because the R7.3 chemistry assesses only 5 nucleotides at a time as they pass through a pore [46]. The coverage per base in each dataset agrees with the results displayed in Fig 1. The multi-modal distributions observed in the MinION data, and not the MiSeq data, is likely due to residual PCR primers. These abundant reads may be attributed to at least one of three factors. First, the two MinION sequences are full-length amplicons and should provide two-fold coverage in these regions. Second, these regions have a much higher abundance of forward strand alignments, which are likely from products of failed extensions and/or early termination caused by the annealing from the other primer set. Third, these smaller products are not enriched on the MiSeq because the tagmentation reaction must integrate in at least two sites in order to sequence the molecule (Fig 3, S2 and S3 Figs and Fig 4).

### Mixture Evaluation

**Fig 5:**
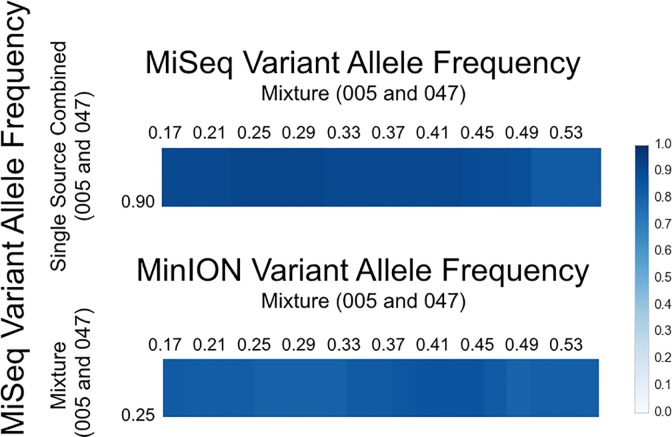
Mixture Concordance by VAF. The MiSeq single source 0.90 VAF call sets were combined for both 005 and 047 to inform the optimal MiSeq mixture comparison. The y-axis has MiSeq VAF SNP call sets for the single source (top) and mixture (bottom). On the top, the MiSeq mixture SNP call sets are plotted by VAF from 0.17 to 0.53 using increments of 0.02 across the x-axis and compared to the single source combined 0.90 VAF SNP set. The F1-Scores are equal between 0.23 and 0.29 in the mixture. On the bottom, the empirically determined VAF range (0.23 to 0.29) for the mixture MiSeq VAF permitted the MiSeq Mixture VAF to be set at 0.25 to compare with MinION VAFs ranging 0.17 to 0.53 using increments of 0.02. The highest F1-Score between the MiSeq (0.25) and MinION (0.45) VAFs are shown in Fig 4.

A 1:1 mixture comprised of two individuals used in the single source evaluation (005 and 047) was analyzed using both the MiSeq and MinION platforms. The combined single source 0.90 VAF MiSeq truth sets for 005 and 047 were used to explore a spectrum of possible detection VAFs in the MiSeq mixture (Fig 5). The MiSeq mixture VAF call sets were the same between 0.23 to 0.29 across the output and contained a single false negative, which occurred at np 4,736 (with a frequency of 0.16 alternate (or C) reads; see Tables 1 and 2), but was identified correctly by the MinION, and was not used in the concordance calculations (Fig 4). Additionally, higher MiSeq mixture VAFs suffered from excluding true positives. It is worth noting that at a mixture VAF of 0.25, the false negative in HVRI at np 16,183 in the single source data is subsequently identified in the mixture. The reason for observing the SNP in the mixture is that the overall detection threshold (VAF) for a mixture must be lower than single source. In theory, a SNP should at most contribute half of the reads at any given locus and would be exactly half of that of a single source experiment. A private SNP should be represented by half of the reads and a shared SNP would be represented by all of the reads. Therefore, it should be no surprise that a shared SNP is far more likely to be detected alongside the obligate decrease in the VAF threshold. The F1-scores across all VAFs are similar until reaching 0.45 for the MiSeq to MiSeq comparison (Fig 5). The 0.25 MiSeq mixture call set was then used as the ground truth for the comparison with the MinION, where the two platforms showed the highest concordance between 0.39 and 0.43 for the MinION. As expected, the concordance in the mixture is slightly lower, Recall: 0.796, Precision: 0.972, and F1-Score: 0.875 (TP: 35, FP: 1, FN: 9), with an even higher incidence of false negatives when compared to the single source samples and a single false positive once again was observed at the same locus (np 2,130–2,135) in the single source 005. It is worth noting that the optimal VAFs presented here are subject to change when considering other mixture ratios (or individuals), as the exact thresholds are not the focus of these experiments.

**Table 1:** SNP Read Counts in Two Loci. Read counts for each of the SNPs shown in Fig 6, which correspond to the six tracks. Counts of the four SNPs in np 3,800–4,800.

	3849 np	4553 np	4736 np	4769 np
MinION:Mixture	A: 146 (43%)	T: 207 (54%)	T: 220 (57%)	A: 49 (13%)
(005 and 047)	G: 180 (53%)	C: 147 (38%)	C: 150 (39%)	G: 313 (84%)
	Other: 14 (4%)	Other: 30 (8%)	Other: 17 (4%)	Other: 11 (3%)
MiSeq: Mixture	A: 1744 (49%)	T: 866 (52%)	T: 951 (84%)	A: 6 (<1%)
(005 and 0047)	G: 1816 (51%)	C: 771 (47%)	C: 178 (16%)	G: 948 (98%)
	Other: 19 (<1%)	Other: 22 (1%)	Other: 6 (<1%)	Other: 12 (1%)
MinION: 005	A: 4 (4%)	T: 92 (84%)	T: 95 (88%)	A: 13 (13%)
(Phased)	G: 88 (93%)	C: 8 (7%)	C: 8 (7%)	G: 87 (85%)
	Other: 3 (3%)	Other: 10 (9%)	Other: 5 (5%)	Other: 2 (2%)
MiSeq:005	A: 12 (1%)	T: 866 (97%)	T: 951 (100%)	A: 3 (<1%)
(Deconvolved)	G: 1816 (98%)	C: 6 (1%)	C: 0 (0%)	G: 830 (99%)
	Other: 17 (1%)	Other: 21 (2%)	Other: 4 (<1%)	Other: 9 (1%)
MinION: 047	A: 94 (88%)	T: 28 (24%)	T: 35 (31%)	A: 12 (11%)
(Phased)	G: 10 (9%)	C: 84 (74%)	C: 73 (64%)	G: 100 (88%)
	Other: 3 (3%)	Other: 2 (2%)	Other: 6 (5%)	Other: 1 (1%)
Miseq: 047	A: 2991 (100%)	T: 3 (<1%)	T: 3 (1%)	A: 1 (<1%)
(Deconvolved)	G: 15 (<1%)	C: 1720 (100%)	C: 354 (99%)	G: 825 (100%)
	Other: 0 (0%)	Other: 0 (0%)	Other: 0 (0%)	Other: 1 (<1%)

**Table 2:** SNP Read Counts in Two Loci. Read counts for each of the SNPs shown in Fig 6, which correspond to the six tracks. Counts of the three SNPs present in np 11,200–11,500.

	11,197 np	11,365 np	11,467 np
MinION:Mixture	T: 100 (43%)	T: 103 (56%)	A: 35 (26%)
(005 and 047)	C: 124 (54%)	C: 71 (39%)	G: 167 (76%)
	Other: 7 (3%)	Other: 9 (5%)	Other: 17 (8%)
MiSeq: Mixture	T: 1097 (46%)	T: 1388 (51%)	A: 48 (1%)
(005 and 0047)	C: 1233 (52%)	C: 1344 (49%)	G: 3202 (98%)
	Other: 47 (2%)	Other: 6 (<1%)	Other: 27 (<1%)
MinION: 005	T: 65 (89%)	T: 17 (26%)	A: 9 (12%)
(Phased)	C: 8 (11%)	C: 46 (71%)	G: 60 (81%)
	Other: 0 (0%)	Other: 2 (3%)	Other: 5 (7%)
MiSeq:005	T: 1097 (95%)	T: 0 (0%)	A: 43 (2%)
(Deconvolved)	C: 13 (1%)	C: 1344 (99%)	G: 2598 (98%)
	Other: 44 (4%)	Other: 5 (<1%)	Other: 18 (<1%)
MinION: 047	T: 7 (8%)	T: 51 (85%)	A: 13 (17%)
(Phased)	C: 71 (89%)	C: 4 (7%)	G: 55 (72%)
	Other: 2 (3%)	Other: 5 (8%)	Other: 8 (11%)
Miseq: 047	T: 5 (<1%)	T: 1388 (98%)	A: 33 (1%)
(Deconvolved)	C: 1233 (97%)	C: 17 (1%)	G: 2682 (98%)
	Other: 30 (2%)	Other: 5 (<1%)	Other: 23 (1%)

### Phasing, Deconvolution, and Assembly

**Fig 6:**
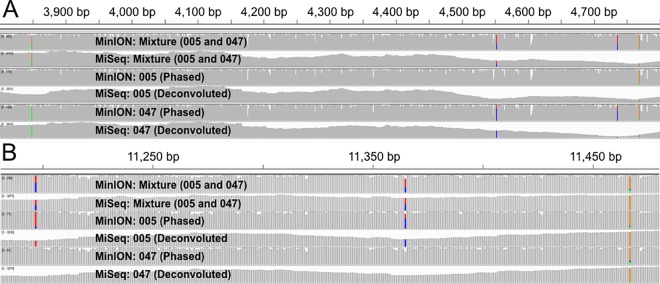
SNP Phasing and Deconvolution in Two Loci. The selected loci contain proximate SNPs that are private to 005 and 047. A) Four SNPs in a 1kb window with ticks of 50 bp (np 3,800–4,800). B) Three SNPs in 300bp window with 50 bp ticks (np 11,200–11,500). Read counts for each of the SNPs are shown In Table 1. MiSeq and MinION mixture alignments are the top two tracks. Phasing and deconvoluted read sets make up the middle and bottom sets of tracks. The middle two tracks are the reads that represent 005 and the bottom two tracks are the reads that represent 047.

**Fig 7:**
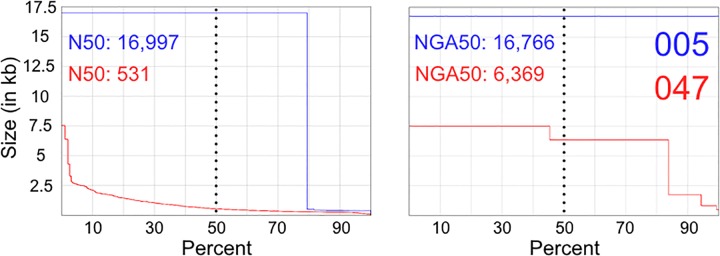
Assembly Statistics. The two assemblies, 005 (blue) and 047 (red), are depicted in each plot where the x-axis is percent of contigs and the y-axis is size in kilobases (kb). A) Plot of the Nx where the dotted line is the N50. B) Plot of the NGAx where the dotted line is NGA50.

The MinION reads that ostensibly spanned the full-length amplicons were capable of being phased due to the digital nature of the data, which typically is not feasible with Sanger sequencing. The phased reads provided high enough accuracy (Table 1) to visually distinguish provenance in the mixture (see Fig 6). The MiSeq reads could then be deconvoluted with the *a priori* knowledge of data from the phased MinION and single source MiSeq reads (Fig 6 and Table 1). Integrating both the phased (MinION) and deconvolved (MiSeq) reads, two distinct assemblies were made, which were impressively 100% concordant with previously described variants [40] for SNPs and INDELs when applying a VAF of 0.75 (S1 Table). The assembly statistics (Fig 7) reveal that a single contig is constructed for 005 and a collection of contigs (ranging several kb) represent 047. The complete set of aligned contigs for both assemblies contained no gaps across the entire length of the mitochondrial genome. The phasing and assembly of mixture reads demonstrates the potential of identifying the composition of a mixture.

## Conclusion

This unbiased and cursory comparison was attempted to assess how the MinION performs relative to MiSeq when sequencing mitochondrial genomes from single sources and mixtures of individuals. Often, a mixture of two individuals can be easier to deconvolve due to the individual donors having different haplogroups and distinguishable variants. In this study, a 1:1 mixture of individuals from the same haplogroup was selected because it is a more challenging mixture to deconvolve with typical forensics workflows. Because of the difficulty in analyzing these types of mixtures, the proof-of-concept phasing of variants using the MinION was successful. Based on these analyses, the MinION has the ability to genotype SNPs on the mitochondrial genome with relatively high precision and recall for single source samples. However, it suffers some loss in detection ability when analyzing mixtures. False negatives occur far more frequently than false positives and usually occur in homopolymeric regions, which is a common issue with some other platforms [44]. However, phasing the long reads generated by the MinION in a mixture can provide physical linkage information about SNPs that is inaccessible with shorter read technologies, allowing in this study differentiation between two individuals of the same haplogroup. Moreover, this study shows that it is possible to integrate mixture data for assembling the entire mitochondrial genome of the contributing individuals to that mixture. Lastly, sequencing of mitochondrial DNA often is used on samples that are highly degraded and contain little or no nuclear DNA, such as unidentified human remains. Therefore, these types of samples will not have sufficiently long fragments to take advantage of the MinION for phasing. However, since mitochondrial DNA tends to persist longer than nuclear DNA, there may be novel sample types, e.g., touch DNA, to consider where mitochondrial DNA may not be so degraded. The MinION could be an extremely useful tool to investigate what types of samples contain relatively intact molecules and characterize mitochondrial DNA degradation lengths from different sample types to potentially extend the value of mitochondrial DNA for human identity testing.

## Materials and Methods

### Sample Preparation

Genomic DNA from individual samples (004, 005, 047) was extracted from whole-blood, previously described by King et al. [40]. All samples were collected anonymously according to University of North Texas Health Science Center’s Institutional Review Board. Samples 004, 005, and 047 were selected for this study because they share the same major haplogroup clade assignment (i.e., 004 is U5a1a1d, and 005 and 0047 are U2e1a1) [40]. The quantity of recovered DNA was determined using the Qubit® dsDNA BR Assay Kit on the Qubit® 2.0 Fluorometer (Life Technologies, Foster City, CA, USA). Samples were normalized to 0.1 ng/μL in molecular grade water, prior to amplification.

### Target Enrichment

The whole mitochondrial genome of each sample was enriched by generating two overlapping amplicons, ~8.3 kb and ~8.6 kb, by long-PCR amplification using the TaKaRa LA PCR Kit (TaKaRa Bio, Otsu, Shiga, Japan), following the protocol previously described in King et al. [40]. The primers used for long-PCR amplification, H8982/L644 and H877/L8789, were described by Gunnarsdóttir et al. [39]. The quantity of PCR product was determined using the Qubit® dsDNA BR Assay Kit on the Qubit® 2.0 Fluorometer. Samples were normalized to 0.5 ng/μL. Amplicon fragment size was evaluated using the Agilent High Sensitivity DNA kit and Agilent 2100 Bioanalyzer (Agilent Technologies, Santa Clara, CA, USA).

### MiSeq Library Preparation and Sequencing

Amplified product of samples 004, 005 and 047 were normalized to 0.2 ng/μL, and the latter two samples were mixed 1:1. Samples 005 and 047 were selected for the mixture study because they share the same haplogroup assignment (U2e1a1) [40]. Library preparation and sequencing were performed using the Nextera XT DNA Library Preparation Kit (Illumina, San Diego, CA, USA) and MiSeq Reagent Kit v2 using a read length of 2 X 250bp, respectively, as described previously [40].

### MinION Library Preparation and Sequencing

Amplified product of samples 004, 005 and 047 were purified using the Qiagen QIAquick PCR Purification Kit (Qiagen, Hilden, Germany), per manufacturer’s instructions. Samples were analyzed using the MinION R7.3 chemistry (FLO-MAP003). Library preparation was carried out using the manufacturer’s instructions for amplicon sequencing using 1 μg of total input DNA (0.5 μg each of two amplicons from single contributors or 0.25 μg each for each amplicon from two-person mixtures). Flow cells were run for approximately 24 hours in total including “topping up” once 16 hours into the run. Basecalling was performed in Metrichor using versions 1.69 (sample 004) and 1.99 (all other samples).

### Data Analysis

Poretools [47] version 0.5.1 was used to generate fastq files for the MinION 2D reads that passed the default quality filters of Metrichor. The MiSeq reads were downloaded from BaseSpace as fastq files. BWA MEM [48] version 0.7.12 was used to align the data to the full reference (1000 genomes hg 19 build 37) genome with–x ont2d mode for the MinION reads and MEM in default mode for the MiSeq reads. Reads that did not align to the mitochondria reference were discarded. Coverage depth was calculated using BEDTools [49] version 2.23.0.

### SNP Detection and Concordance

For single source samples, variants were called on the mitochondria aligned data with Freebayes [50] version 0.9.21 with a ploidy of 1. A set of *M x N* comparisons were made where the sets *M* (MiSeq) and *N* (MinION) both contain 19 SNP subsets comprised of *m*_*0*.*05*_, *m*_*0*.*10*_, *…*, *m*_*0*.*95*_ and *n*_*0*.*05*_, *n*_*0*.*10*_, *…*, *n*_*0*.*95*_ ranging the VAF (-F Freebayes option) from 0.05 to 0.95 in increments of 0.05 for each platform. Each SNP subset was filtered in the following way: variants were normalized and both biallelic block substitutions and multi-allelic variant calls were decomposed into individual calls using vt [51] version 0.5, where the resulting calls were then required to be classified as a SNP and having a quality score above 20, even though the MinION data do not appear to follow the traditional phred scale [45].

Mixture samples were analyzed in a similar manner. The only difference in approach was using a ploidy of 2 when running Freebayes and how the truth sets were generated. A set of *M* (MiSeq) call sets comprised of *m*_*0*.*15*_, *m*_*0*.*17*_, *…*, *m*_*0*.*53*_ subsets with varying allele from 0.17 to 0.53 in increments of 0.02 were made. A set *N* with a single member *n*_*0*.*90*_ was made using the combined truth sets from the 0.90 VAF single source calls sets from 005 and 047. The call set *n*_*0*.*90*_ contained 31 shared SNPs and 12 private SNPs between 005 and 047 (the false negative at np 16,183 was not detected in either call set). The MiSeq mixture VAFs from 0.23 to 0.29 were all equivalent call sets, where np 4736 is a false negative private for sample 047; however, a previously false negative shared SNP at 16,183 is detected in the mixture (S1 Table and 38). The MiSeq VAF of 0.25 was then used to assess concordance with the MinION. A set of *M* (MinION) call sets comprised of *m*_*0*.*15*_, *m*_*0*.*17*_, *…*, *m*_*0*.*53*_ subsets with VAFs from 0.17 to 0.53 in increments of 0.02 were made to compare against the set N with single member *n*_*0*.*25*_ of the MiSeq Mixture call set. All calculations for recall, precision, and F1-scores were made using the below definitions, and concordance was plotted using Circos [52] version 0.69.

$$Recall\;:\;\frac{m⋂n}{(m⋂n)⋃\left(m-n\right)}\;or\;\frac{TP}{\left(TP+FN\right)}$$

$$Precision\;:\;\frac{m⋂n}{(m⋂n)⋃\left(n-m\right)}\;or\;\frac{TP}{\left(TP+FP\right)}$$

$$F_{1}-Score\;:\;2\frac{\left(Precision\;*\;Recall\right)}{\left(Precision+Recall\right)}$$

SNPs were assessed as True Positives, False Negatives, and False Positives at each site using the various MiSeq SNP sets as the ground truth for these comparisons.

### Phasing, Deconvolution and Hybrid Assembly

MinION reads covering the two full-length amplicons were extracted by separating bam records that intersected the bed interval (zero-based half-open) of 1000 to 8000 and an interval of 10,000 to 15,000. These reads were required to exceed 8000 bases to capture only the reads from the fully extended amplicon. Phasing was performed with SAMtools [53] phase version 0.1.19 on the two sets of extracted records.

MiSeq read pairs were extracted and assigned from the MiSeq bam file using a combination of BEDTools and JVarkit git commit 865252a [49, 54] at the private variants (12 SNPs and 2 INDELs) in the single contributor datasets (S1 Table). The read pairs were sorted into three pools of extracted reads, being shared, private to 005, or private to 047 based on the full variant set in S1 Table. JVarkit was used to generate alignment bases relative to the reference offset and cigar operations. Any read pair that spanned one of the 12 SNP loci was extracted from the bam file and queried for the base relative to the reference alignment position. This base was then compared against the two possible alleles determined by the truth set and assigned to the corresponding individual if the cigar operation was a match (or M). The reads that did not meet these requirements were placed into the pool of shared reads. For the two insertion events, pairs were extracted if they aligned within 10bp on both sides of the insertion site, if they had a cigar operation of insert (or I) they were attributed to sample with an insertion, otherwise the reads were assigned to the other. Thus, all remaining non-extracted bam records were comprised of reads that represented shared genotypes or supported variants in both individuals and the two sets of extracted reads contained the private genotypes of the two contributors in the mixture. The private read pools were then separately added back to the pool of shared reads and made into two sets of deconvoluted MiSeq bams that represent the known genotypes of these individuals. The reads in these bams were then made into of fastq files if the pair was mapped and contained no secondary alignments. These high quality MiSeq fastq files were then assembled with the previously phased MinION reads, which were also converted into fastq files with BEDTools bamtofastq [49]. The hybrid assembly (using both MinION and MiSeq data) was performed on each individual using SPAdes [55] with 10 iterations of bayes hammer read correction (-i 10) and aligned with BWA SW [56] with default parametes, and contigs with mapping quality 0 were removed, evaluating the contigs with unique mappings [57]. It should be noted a mapping quality of 20 is a typical heuristic for short-read alignments; however, these alignments were assembled contigs. Contigs generated from amplicon sequencing, which uniquely map back to their original amplicon(s) after extensive error correction, are likely to be high quality. The aligned contigs were visually inspected in IGV [58] version 2.3.75 and a VAF of 0.75 matched the full call set for the mixture (S1 Table).

## Supporting Information

S1 Table Variant Call Sets. The complete set of SNP and INDEL calls presented here are from the previous publication [37]. Sample names 004, 005, 047, and the Mixture are at the top of each column with variant positions and type indicated in each row below.
(DOCX)

S1 Fig Visualization of repetitive loci. Two 15bp windows are shown for both MiSeq and MinION alignments for sample 005 using IGV. The VAF used in the coverage track is set to 0.60 to display relative proportions of reads that are below the MiSeq threshold. **A).** The SNP at np 16,183 is excluded from the truth set due to poor alignment and length heteroplasmy of repetitive Cs in the locus. **B)** The repetitive sequence proceeding np 310 contains an insertion of a C that causes a false positive SNP call when the VAF is adjusted too low.
(TIFF)

S2 Fig Coverage and Concordance Circos plot for 005. The coverage depth per base is shown for the MiSeq (orange) and MinION (blue) shown on the outer ring using a log_10_ scale. The inner ring contains the per SNP concordance using a VAF of 0.90 for the MiSeq and 0.65 VAF for the MinION. The text color denotes the categorization of each SNP using a VAF combination providing the highest concordance: green text indicates that the SNP was a true positive, black text is for false negatives, and red is for false positives in the MinION call sets. An asterisk (*) means the SNP was not observed with the MiSeq VAF and not used for calculating recall, precision, and the F1-Score. Black arrows indicate the locations and orientations of the primers used for amplification.
(TIF)

S3 Fig Coverage and Concordance Circos plot for 047. The coverage depth per base is shown for the MiSeq (orange) and MinION (blue) shown on the outer ring using a log_10_ scale. The inner ring contains the per SNP concordance using a VAF of 0.90 for the MiSeq and 0.65 VAF for the MinION. The text color denotes the categorization of each SNP using a VAF combination providing the highest concordance: green text indicates that the SNP was a true positive, black text is for false negatives, and red is for false positives in the MinION call sets. An asterisk (*) means the SNP was not observed at with the MiSeq VAF and not used for calculating recall, precision, and the F1-Score. Black arrows indicate the locations and orientations of the primers used for amplification.
(TIF)
