# Supplementary material for: A Comparison and Integration of MiSeq and MinION Platforms for Sequencing Single Source and Mixed Mitochondrial Genomes
Source: PLoS One. 2016 Dec 9;11(12):e0167600. doi: 10.1371/journal.pone.0167600 (PMC5147911; doi:10.1371/journal.pone.0167600)
Supplement: S1 Table — The complete set of SNP and INDEL calls presented here are from the previous publication [37]. Sample names 004, 005, 047, and the Mixture are at the top of each column with variant positions and type indicated in each row below. (DOCX) [file pone.0167600.s001.docx]

**Table S1. Variant Call Sets.**

| 004 | 005 | 047 | Mixture |
| --- | --- | --- | --- |
| 73G | 73G | 73G | 73G |
|  | 152C | 152C | 152C |
| 185A |  |  |  |
|  | 217C | 217C | 217C |
| 204C |  |  |  |
| 263G | 263G | 263G | 263G |
|  | 309.1C† | 309.1C† | 309.1C† |
| 315.1C† | 315.1C† | 315.1C† | 315.1C† |
|  | 340T |  | 340T |
|  | 508G | 508G | 508G |
|  | 524.1AC† |  | 524.1AC† |
| 750G | 750G | 750G | 750G |
| 1438G | 1438G | 1438G | 1438G |
| 1700C |  |  |  |
|  | 1811G | 1811G | 1811G |
| 2706G | 2706G | 2706G | 2706G |
|  | 3116T |  | 3116T |
| 3197C |  |  |  |
|  | 3720G | 3720G | 3720G |
|  |  | 3849A | 3849A |
|  |  | 4553C | 4553C |
|  |  | 4736C | 4736C* |
| 4769G | 4769G | 4769G | 4769G |
|  | 5390G | 5390G | 5390G |
|  | 5426C | 5426C | 5426C |
| 5495C |  |  |  |
|  | 6045T | 6045T | 6045T |
|  | 6152C | 6152C | 6152C |
| 7028T | 7028T | 7028T | 7028T |
|  |  | 8473C | 8473C |
| 8860G | 8860G | 8860G | 8860G |
| 9477A |  |  |  |
|  | 10754G |  | 10754G |
|  | 10876G | 10876G | 10876G |
|  | 11197T |  | 11197T |
|  | 11365C |  | 11365C |
| 11467G | 11467G | 11467G | 11467G |
| 11719A | 11719A | 11719A | 11719A |
|  | 11732C |  | 11732C |
| 12308G | 12308G | 12308G | 12308G |
| 12372A | 12372A | 12372A | 12372A |
|  |  | 12557T |  |
|  | 13020C | 13020C | 13020C |
| 13617C |  |  |  |
|  | 13734C | 13734C | 13734C |
| 14766T | 14766T | 14766T | 14766T |
| 14793G |  |  |  |
| 15218G |  |  |  |
| 15326G | 15326G | 15326G | 15326G |
|  | 15907G | 15907G | 15907G |
| 15924G |  |  |  |
|  | 16051G | 16051G | 16051G |
|  |  | 16092C | 16092C |
|  | 16129C | 16129C | 16129C |
|  | 16183C* | 16183C* | 16183C |
|  | 16189C | 16189C | 16189C |
|  |  | 16193.1C† |  |
| 16256T |  |  |  |
| 16270T |  |  |  |
| 16362C | 16362C | 16362C | 16362C |
| 16399G |  |  |  |
|  | 16519C | 16519C | 16519C |

Footnote: A dagger (†) denotes INDELs and an asterisk (*) is a putative false negative in the MiSeq call set for this study. Variants marked with either symbol were not used in the platform concordance analyses. The MiSeq call sets presented in this study (when compared to King et al. [37]) had minimal read filtering and a global variant allele frequency cutoff applied to present a platform agnostic assessment.
