# Supplementary figures and images for: A Comparison and Integration of MiSeq and MinION Platforms for Sequencing Single Source and Mixed Mitochondrial Genomes
Source: PLoS One. 2016 Dec 9;11(12):e0167600. doi: 10.1371/journal.pone.0167600 (PMC5147911; doi:10.1371/journal.pone.0167600)

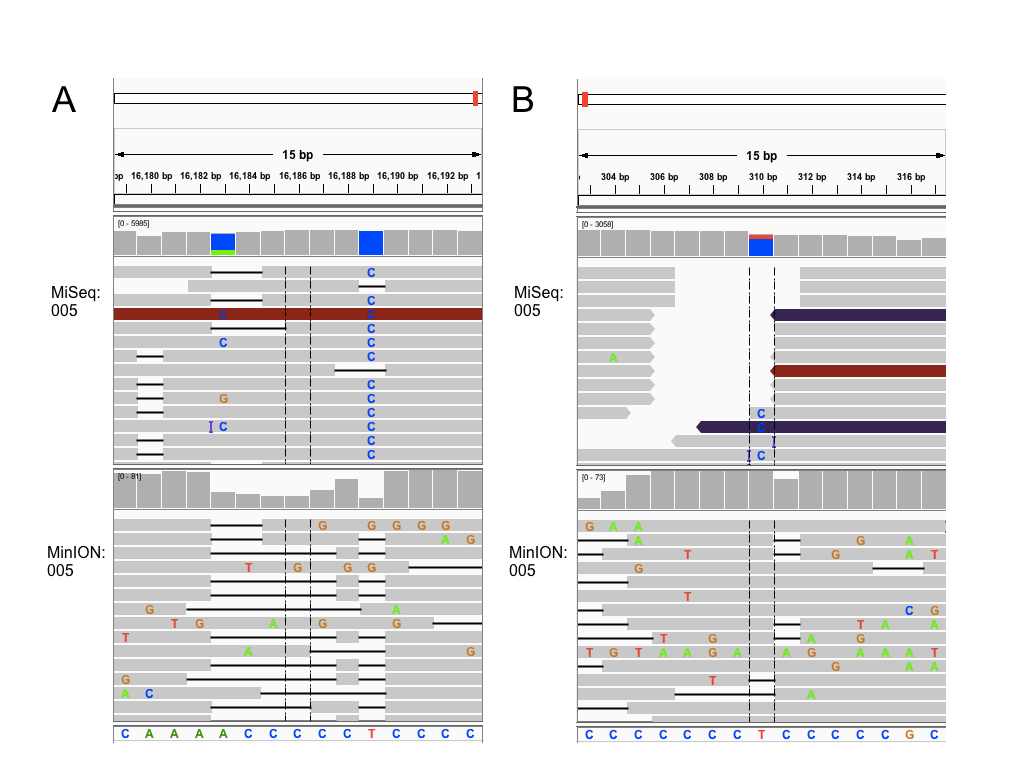

Supplement: S1 Fig — Two 15bp windows are shown for both MiSeq and MinION alignments for sample 005 using IGV. The VAF used in the coverage track is set to 0.60 to display relative proportions of reads that are below the MiSeq threshold. A). The SNP at np 16,183 is excluded from the truth set due to poor alignment and length heteroplasmy of repetitive Cs in the locus. B) The repetitive sequence proceeding np 310 contains an insertion of a C that causes a false positive SNP call when the VAF is adjusted too low. (TIFF) [file pone.0167600.s002.tiff]

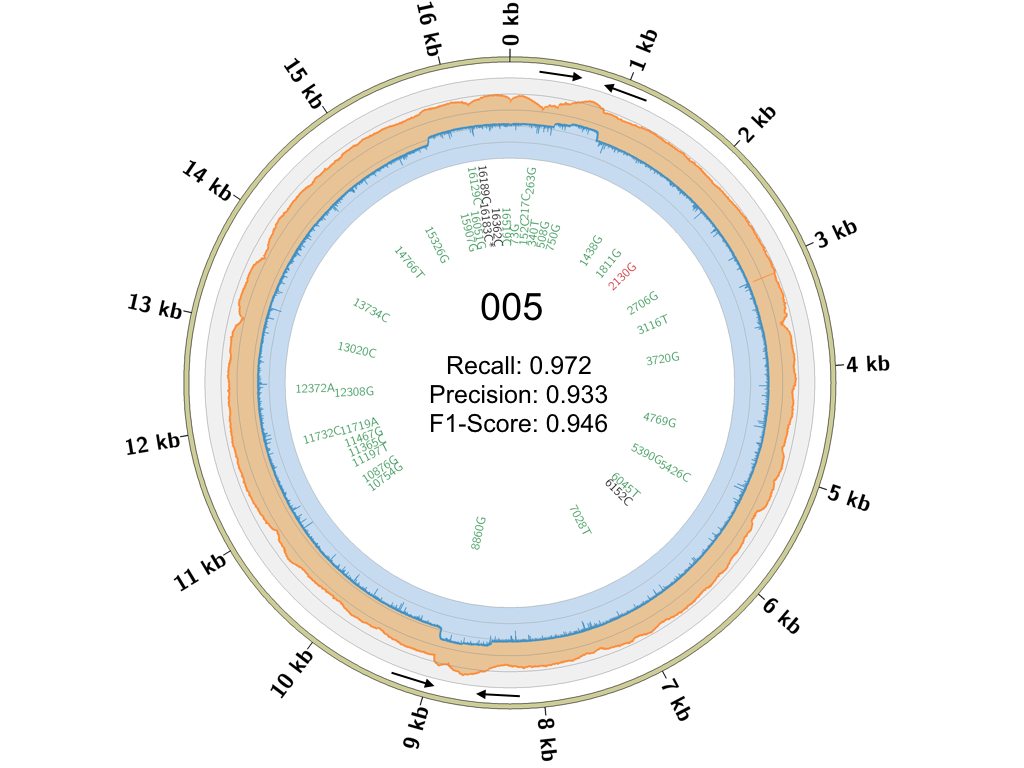

Supplement: S2 Fig — The coverage depth per base is shown for the MiSeq (orange) and MinION (blue) shown on the outer ring using a log10 scale. The inner ring contains the per SNP concordance using a VAF of 0.90 for the MiSeq and 0.65 VAF for the MinION. The text color denotes the categorization of each SNP using a VAF combination providing the highest concordance: green text indicates that the SNP was a true positive, black text is for false negatives, and red is for false positives in the MinION call sets. An asterisk (*) means the SNP was not observed with the MiSeq VAF and not used for calculating recall, precision, and the F1-Score. Black arrows indicate the locations and orientations of the primers used for amplification. (TIF) [file pone.0167600.s003.tif]

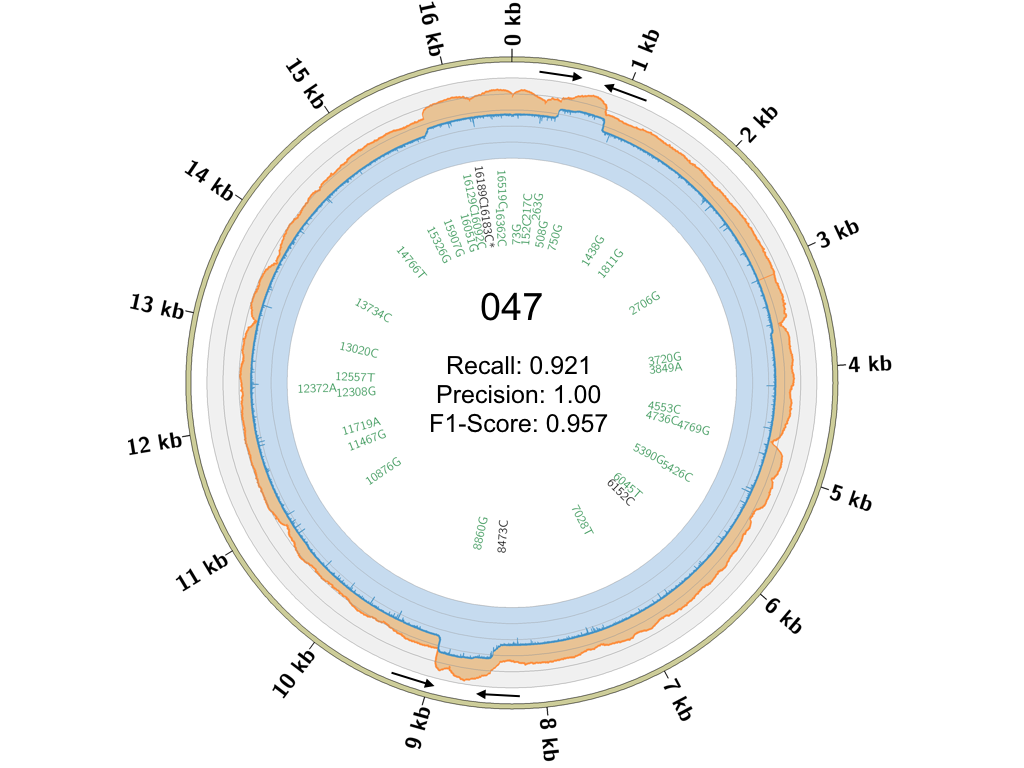

Supplement: S3 Fig — The coverage depth per base is shown for the MiSeq (orange) and MinION (blue) shown on the outer ring using a log10 scale. The inner ring contains the per SNP concordance using a VAF of 0.90 for the MiSeq and 0.65 VAF for the MinION. The text color denotes the categorization of each SNP using a VAF combination providing the highest concordance: green text indicates that the SNP was a true positive, black text is for false negatives, and red is for false positives in the MinION call sets. An asterisk (*) means the SNP was not observed at with the MiSeq VAF and not used for calculating recall, precision, and the F1-Score. Black arrows indicate the locations and orientations of the primers used for amplification. (TIF) [file pone.0167600.s004.tif]
